# Characterizing the Influence of a Heterotrophic Bicosoecid Flagellate *Pseudobodo* sp. on the Dinoflagellate *Gambierdiscus balechii*

**DOI:** 10.3390/toxins15110657

**Published:** 2023-11-14

**Authors:** Xiaowan Liu, Yihan Ma, Jiajun Wu, Pengbin Wang, Yinuo Wang, Anli Wang, Qizhao Yin, Haiying Ma, Leo Lai Chan, Bin Wu

**Affiliations:** 1 State Key Laboratory of Marine Pollution, Department of Biomedical Sciences, City University of Hong Kong, Hong Kong SAR 999077, China; xiaowliu5-c@my.cityu.edu.hk (X.L.); jiajunwu@cityu.edu.hk (J.W.); haiyingma2-c@my.cityu.edu.hk (H.M.); 2 Ocean College, Zhejiang University, Zhoushan 321000, China; mayihan@zju.edu.cn (Y.M.); wangyinuo@zju.edu.cn (Y.W.); anliwang@zju.edu.cn (A.W.); qizhaoyin@zju.edu.cn (Q.Y.); 3 Shenzhen Key Laboratory for the Sustainable Use of Marine Biodiversity, Research Centre for the Oceans and Human Health, City University of Hong Kong Shenzhen Research Institute, Shenzhen 518057, China; 4 Key Laboratory of Marine Ecosystem Dynamics, Second Institute of Oceanography, Ministry of Natural Resources, Hangzhou 310012, China; algae@sio.org.cn; 5 The Fourth Institute of Oceanography, Ministry of Natural Resources, Beihai 536000, China

**Keywords:** microbial interaction, *Gambierdiscus balechii*, heterotrophic bicosoecid flagellate, *Pseudobodo* sp., gambierones

## Abstract

Microbial interactions including competition, mutualism, commensalism, parasitism, and predation, which can be triggered by nutrient acquisition and chemical communication, are universal phenomena in the marine ecosystem. The interactions may influence the microbial population density, metabolism, and even their environmental functions. Herein, we investigated the interaction between a heterotrophic bicosoecid flagellate, *Pseudobodo* sp. (Bicoecea), and a dinoflagellate, *Gambierdiscus balechii* (Dinophyceae), which is a well-known ciguatera food poisoning (CFP) culprit. The presence of *Pseudobodo* sp. inhibited the algal proliferation and decreased the cardiotoxicity of zebrafish in the algal extract exposure experiment. Moreover, a significant difference in microbiome abundance was observed in algal cultures with and without *Pseudobodo* sp. Chemical analysis targeting toxins was performed by using liquid chromatography-tandem mass spectrometry (LC-MS/MS) combined with molecular networking (MN), showing a significant alteration in the cellular production of gambierone analogs and some super-carbon chain compounds. Taken together, our results demonstrated the impact of heterotrophic flagellate on the photosynthetic dinoflagellates, revealing the complex dynamics of algal toxin production and the ecological relationships related to dinoflagellates in the marine environment.

## 1. Introduction

Dinoflagellates are one of the most significant fundamental components of aquatic ecosystems, serving as important primary producers and grazers, and playing a key role in aquatic food webs [1]. However, dinoflagellates attracted widespread attention because of producing harmful algal blooms (HABs), resulting in the death of fish and other marine animals. Moreover, these HABs have been linked to various types of human illness caused by consuming contaminated seafood, which include paralytic shellfish poisoning (PSP), neurotoxic shellfish poisoning (NSP), amnesic shellfish poisoning (ASP), diarrheic shellfish poisoning (DSP), and ciguatera fish poisoning (CFP) [2]. Over the past few decades, abiotic and biotic factors involved in the formation and extinction of HABs and mechanisms of toxin production by dinoflagellates have increasingly attracted wide attention from scientists around the world [3]. Among these, the microorganism living in the phycosphere of dinoflagellates is one of the most significant factors. They have been reported to constantly interact with dinoflagellates, profoundly affecting algal physiology and even toxin production. For instance, a series of algicidal bacteria, including *Cytophaga* sp., *Flavobacterium* sp., *Bacteroidetes* sp., and *Vibrio* sp., can cause cell lysis of dinoflagellates by direct or indirect mode of attack [4,5]. The bacteria *Bacillus* sp. S10 shows inhibitory effects on the growth and toxin production of *Alexandrium tamarense* [6]. Apart from bacteria, heterotrophic heterokonts, using organic matter for energy production, are also common and abundant in the phycosphere of microalgae in nature [7]. There is growing evidence suggesting that heterotrophic heterokonts are the most important pathogen of microalgae, for example, some species of *Amoebophrya* [8], *Perkinsozoa* [9], and *Parvilucifera* [10] have a considerable contribution to mass mortalities of dinoflagellates.

The benthic dinoflagellate genus *Gambierdiscus*, widely distributed in tropical and subtropical regions of the oceans, can produce diverse ladder-shaped polyether toxins including ciguatoxins (CTXs), maitotoxins (MTXs), gambierone, gambierone derivatives, gambierol, gambieroxide, and gambieric acids [11]. These toxins are the causative agents of ciguatera food poisoning (CFP) which is the most common non-bacteria seafood-related illness worldwide [12]. CFP patients may experience symptoms such as numbness of the lips, diarrhea, vomiting, and sensitivity to hot and cold [13]. In severe cases, there may be rapid progression to breathing difficulties, muscular paralysis, low blood pressure, and abnormally slow heartbeat [14]. Unfortunately, there is no effective treatment or antidote for CFP patients and even cooking cannot eliminate these toxins from contaminated seafood [15]. Moreover, climate change and human-induced fertilization in coastal oceanographic and ecological systems have led to an increase in the frequency and severity of HABs in marine ecosystems, posing a significant threat to human health by influencing fisheries and aquaculture [3]. For instance, the CFP incidence has caused an increasingly significant societal impact, with 50,000–500,000 intoxications occurring annually [16]. Therefore, it is important to understand the mechanism underlying the influence of algal proliferation and cellular toxin production in *Gambierdiscus*, in which the phycosphere plays a critical role. Previous studies on the interaction between *Gambierdiscus* and its phycosphere microorganisms have been mainly confined to the microalgae–bacteria interaction [17,18,19]. Apart from bacteria, heterotrophic heterokonts are a crucial part of phycosphere microorganisms, making it reasonable to hypothesize that there is a profound interaction between the dinoflagellate *Gambierdiscus* and pathogenetic heterotrophic heterokonts, which requires further investigation.

It has been reported that pathogenetic heterotrophic heterokonts (bicosoecids) play a crucial role in the carbon cycle of aquatic ecosystems, as they frequently interact with bacteria and phytoplankton [20]. However, there is a lack of information on them because they possess limited morphological features and phylogenetic schemes for identification [21]. *Pseudobodo* is a kind of bicosoecids that are heterokont, bacterivorous, and heterotrophic flagellates that inhabit both marine and freshwater environments [22]. Currently, only two species in the *Pseudobodo* genus have been reported [23]. *P. tremulans* was first reported by Griessmann as early as 1913 [24]. Other studies showed that *Pseudobodo* was capable of phagocytizing bacteria and infected green microalgae [25,26]. Although living in the same scaffold, the influence of *Pseudobodo* on dinoflagellate is still unknown.

To examine the nature of the interaction between the dinoflagellate and pathogenetic heterotrophic heterokonts as we hypothesized, an investigation on the impact of the new pathogen, *Pseudobodo* sp., on *Gambierdiscus balechii* was conducted in this study. Our results showed that the algal proliferation and cardiotoxicity of zebrafish in the algal extract exposure experiment were reduced by the infection of *Pseudobodo* sp. Alterations of the algal-associated bacterial community were also observed. The toxin family, gambierones, was deciphered in *G. balechii* 1123M1M10 by tandem mass spectrometry (MS/MS) analysis coupled with the Global Natural Products Social Molecular Networking (GNPS) tool. The detailed chemical analysis of toxins revealed that the infection of *Pseudobodo* sp. can alter the toxin production of *Gambierdiscus balechii*.

## 2. Results

### 2.1. Morphological Characteristics and Phylogenetic Analysis of Pseudobodo sp.

**Figure 1:**
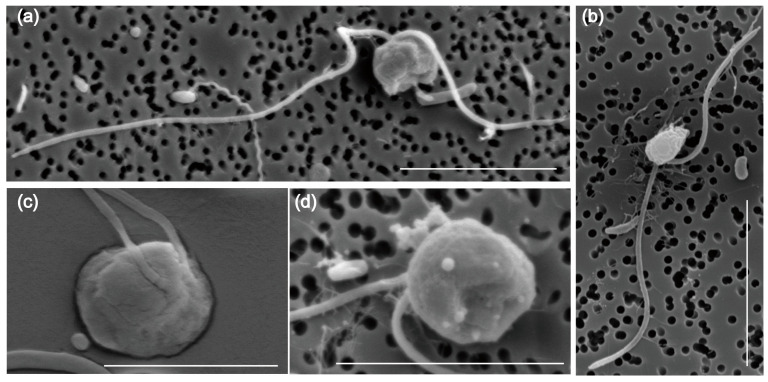
Morphology of *Pseudobodo* sp. under scanning electron microscopy: (**a**) egg-shaped Pseudobodo sp.; (**b**) pear-shaped *Pseudobodo* sp.; (**c**) two flagella emerging from the base of a small apical papilla; (**d**) a fixed hollow on the cell surface. (Scale bars: (**a**,**b**) = 5 μm, (**c**,**d**) = 4 μm.)

The morphology and ultrastructure of the new pathogen *Pseudobodo* sp. were investigated under light microscopy and scanning electron microscopy (SEM). The cells of *Pseudobodo* sp. are pear-shaped or egg-shaped (Figure 1a–d) and have a fixed hollow on the cell surface that is likely the cytostome (Figure 1a,d). Two flagella of unequal length emerge from the base of a small apical papilla (Figure 1a–c), and the smooth posterior passes through a ventral furrow which is used for attachment to substratum or water film (Video S1). In addition, the anterior flagellum does not have mastigonemes (Figure 1a,b), and the body length of *Pseudobodo* sp. is around 2–4 μm.

**Figure 2:**
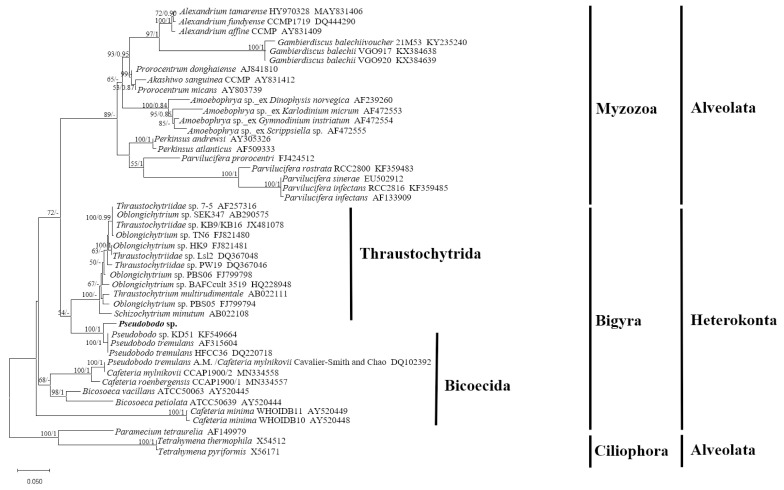
Maximum likelihood tree of the SSU dataset (1416 bp). Paramecium tetraurelia, Tetrahymena thermophila, and Tetrahymena pyriformis were included as the out-group. The best model, chosen by MrModel-Test 2.3, was GTR+I+G. On the nodes are indicated the maximum-likelihood bootstrap values followed by Bayesian posterior probabilities. Only values >50% (ML) and 0.50 (BI) are shown.

The SSU rRNA gene sequence obtained from *Pseudobodo* sp. showed pairwise identities of a 95.41~95.55% to *P. tremulans* sequence, a 95.49% to *Boroka* sp. sequence, and a 93.73~95% to *Bicosoecida* sp. sequence. However, sequence similarity between *Pseudobodo* sp. and other members from the Labyrinthulomycetes, such as *Oblongichytrium* sp. and *Thraustochytriidae* sp., was lower (≤87.73%). The maximum likelihood (ML) tree based on the SSU rRNA gene sequence (Figure 2) showed that the pathogenetic heterokont in this study was placed within the Pseudobodo clade with strong statistical supports for ML value (100) and Bayesian inference (1). All Pseudobodo species were clustered together except Pseudobodo tremulans A. M. (DQ102392), which was reported to be misidentified and was Cafeteria mylnikovii [27].

### 2.2. The Impacts of Pseudobodo sp. on Bacterial Community of Algal Culture

To investigate the influence of *Pseudobodo* sp. on bacterial community, the bacterial abundance in both groups where *Pseudobodo* sp. was absent and present was estimated. We used 16S rRNA metagenomic sequencing to characterize the bacterial community in control and infected groups. The high-throughput sequencing of 6 libraries generated 360,982 reads, which were annotated and classified into 12 phyla. The sample completeness indicator (coverage value) of each treatment group was 1, indicating that the sequencing depth was sufficient to capture all taxa in the bacterial communities [28].

**Figure 3:**
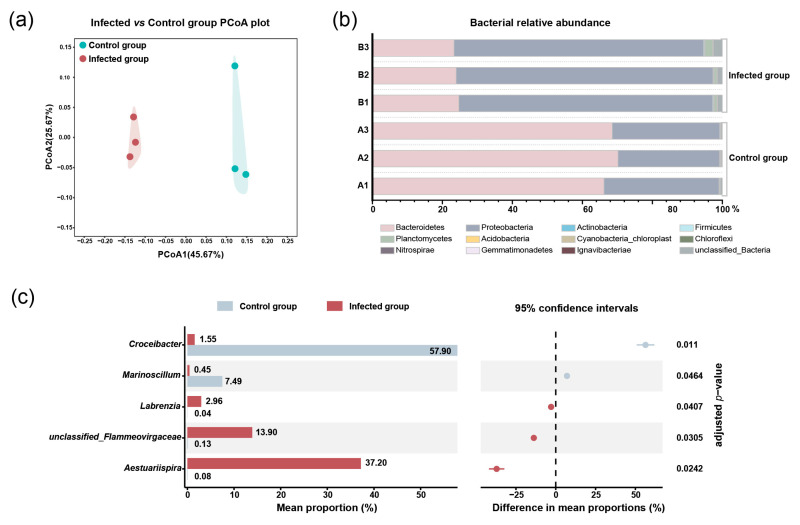
(**a**) PCoA of samples at the OUT level. (**b**) Relative abundance of bacteria at the phylum level. (**c**) The significant difference in bacterial abundance at the genus level.

Principal coordinates analysis (PCoA) showed that three infected groups clustered together and were separated from three clustered control groups (Figure 3a), indicating that the bacterial community was influenced by *Pseudobodo* sp. infection. The results of relative abundance showed that Bacteroidetes and Proteobacteria were dominant phyla in both control and infected groups (Figure 3b). However, when we compared the control and infected groups, the abundance of Bacteroidetes decreased markedly from an average percentage of 68% to 24%, while the Proteobacteria increased from 31% to 72% in the infected group (adjusted *p*-value < 0.05). At the genus level, a significant shift (adjusted *p*-value < 0.05) in the bacterial proportion was observed in five genera (Figure 3c) which belong to the two dominant phyla, including Bacteroidetes phylum: *Croceibacter* sp. and unclassified_*Flammeovirgaceae* sp.; Proteobacteria phylum: *Marinoscillum* sp., *Labrenzia* sp., and *Aestuariispira* sp. We found that the proportion of *Labrenzia* sp., *Aestuariispira* sp., and unclassified_*Flammeovirgaceae* sp. was significantly increased by the infection. On the other hand, *Croceibacter* sp. and *Marinoscillum* sp. were significantly decreased by the infection.

### 2.3. The Alteration of Morphology and Toxicity of G. balechii 1123M1M10 by Infection

**Figure 4:**
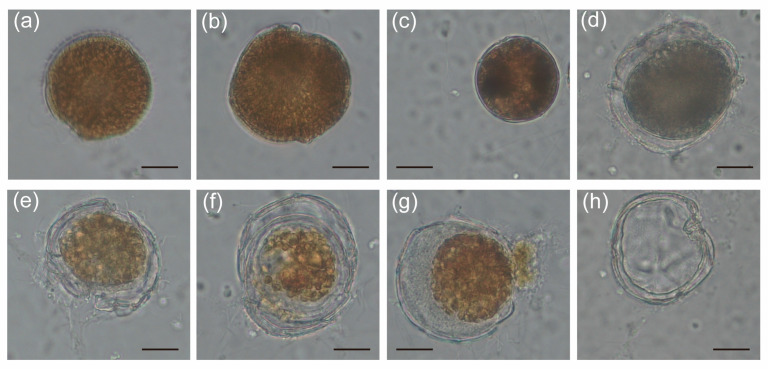
Light microscopy micrographs showing morphological characteristics of infected *G. balechii* 1123M1M10: (**a**) Healthy *G. balechii* 1123M1M10 cell served as control. (**b**) *Pseudobodo* sp. attached to the algal surface. (**c**) *G. balechii* 1123M1M10 cell showed a round body with condensed cytoplasm. (**d**–**h**) The outermost membrane of *G. balechii* 1123M1M10 was broken and algal cytoplasm was released. (Scale bars: (**a**–**h**) = 20 µm.)

To investigate the alteration caused by the infection of *Pseudobodo* sp. on *G. balechii* 1123M1M10, co-culture experiments were carried out. The morphological characteristics of both control and infected groups were monitored by inverted light microscopy (Figure 4a–h). After 28 days of infection, the algal density of the infected groups was lower than that of the control groups (Table S1). From the live observation, many infected phenomena were found, for instance, *Pseudobodo* sp. can attach to the algal surface, the algal cytoplasm was released, and the infected algae were gradually filled with pathogens (Figure 4b–h). We also noticed that dinocyst-like cells exist during the process of infection (Figure 4c).

**Figure 5:**
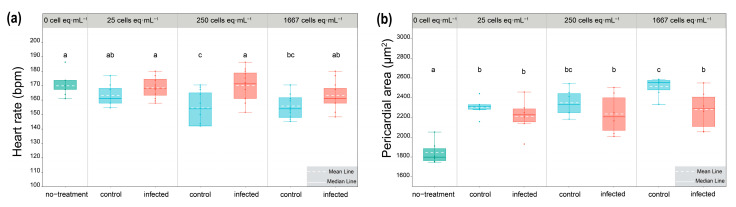
(**a**) Zebrafish heart rate (*n* = 12) at 72 hpf after exposure to control and infected algal extracts. (**b**) Zebrafish pericardial area (*n* = 6) at 72 hpf after exposure to control and infected algal extracts. The superscripts a, b, ab, c, and bc are significant homogenous subsets of means between groups. Boxplots with different superscripts are statistically significant at the 0.05 level by the one-way ANOVA and Duncan’s test. (Boxplot with green color: no-treatment groups; blue color: control groups; red color: infected groups.)

To investigate the variation of algal toxicity caused by infection, the experiment of toxicity assessment was conducted. No embryo survived under the concentration of 8333 cells eq·mL^−1^ for the initial 24 h exposure. The pericardial area and heart rates were measured at 72 hpf when the heart had already formed to function. Heart rates were all significantly reduced in zebrafish exposed to extracts of control groups, as compared to the no-treatment group, while those in zebrafish exposed to extracts of infected groups were not significantly reduced (*p* < 0.05, Figure 5a). Having noticed that pronounced pericardial edema occurred by 72 hpf in all algal extracts exposed to zebrafish, the pericardial areas of zebrafish exposed to each concentration of control and infected groups were measured (Figure 5b). The results showed all extracts significantly enlarged the pericardial areas of zebrafish as compared with the no-treatment groups. A significant difference between the control and infected groups was observed in the concentration of 1667 cells eq·mL^−1^ (Figure 5b). Overall, the infection can reduce cardiotoxicity in the zebrafish model.

### 2.4. Response of Algal Toxin Production to Pseudobodo sp. Infection

**Figure 6:**
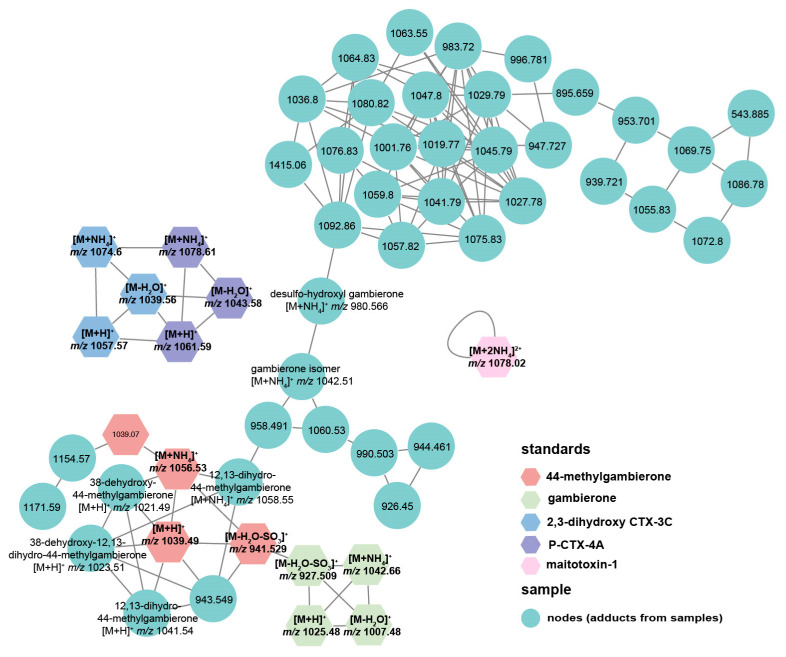
Molecular networks comprised of toxins detected in *G. balechii* 1123M1M10 with coloration based on chemical class.

To investigate the toxin profile of *G. balechii* 1123M1M10, an untargeted metabolomics study was conducted using UHPLC-ESI-Q-TOF-MS/MS combined with the GNPS method. The MS analysis in positive mode yielded more chemical information than in negative mode and was, therefore, initially selected for further investigation. Algal toxins in both groups that Pseudobodo sp. was absent and present were analyzed. Five toxin standards including gambierone, 44-methylgambierone, P-CTX-4A, 2,3-dihydroxy-3C, and maitotoxin 1, together with 12 fragmentation data files from two groups, were used for GNPS analysis. In MN, six reported gambierones [11], two exogenous gambierones standards (gambierone and 44-methylgambierone), along 36 nodes were clustered together (Figure 6), which indicates that these 36 nodes are likely adducts of gambierone analogs since MN is a spectral correlation and visualization approach that can detect sets of spectra from related molecules [29]. By examining their product ion spectra, we found that the nodes including 958.491, 1060.53, 990.503, 944.461, 926.45, 943.549, 1154.57, and 1171.59 showed the same MS^2^ spectrum pattern as gambierones (Figure S1), such as the sulfate loss (*m*/*z* 959 or 945), successive water losses, specific fragments *m*/*z* 233, *m*/*z* 219, *m*/*z* 215, *m*/*z* 193, *m*/*z* 161, *m*/*z* 123, and *m*/*z* 109 [11,30,31], proving that these nodes are the adducts of gambierone analogs. Although the MS^2^ spectra of other nodes showed successive water losses, there were no specific fragments observed, indicating that they are the adducts of a kind of polyol-polyene super-carbon chain compound [32]. Two CTX standards were clustered without any connection with other nodes, indicating that there are no detected CTXs in these samples (Figure 6). Moreover, MTXs were also not found by the GNPS approach because there were no nodes connecting with the MTX-1 standard (Figure 6).

**Figure 7:**
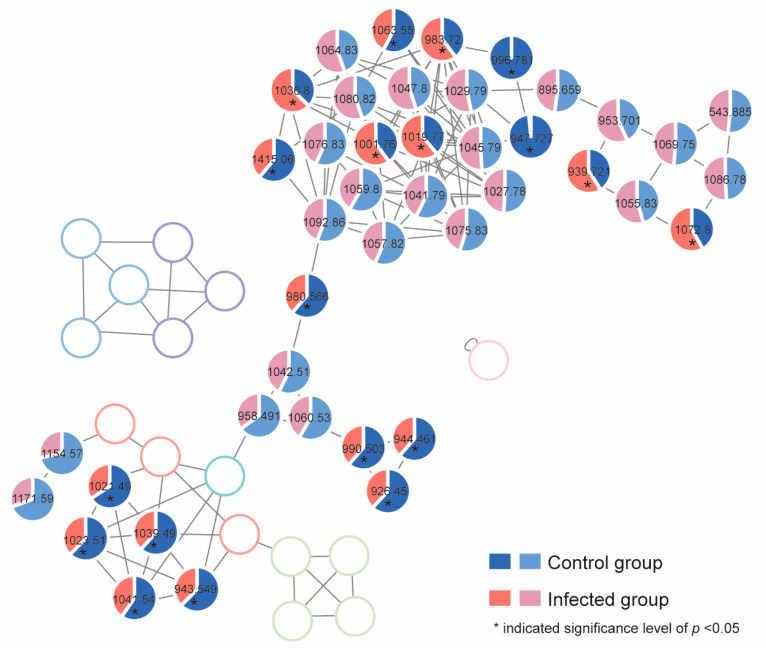
The mean proportion of each detected toxin in both the control and infected algal groups by using UHPLC-ESI-Q-TOF-MS/MS. (The blank pie charts represent the nodes that were not detected in samples or were calculated using other type of adducts.)

The alteration of the cellular production level of the gambierones and polyol-polyene super-carbon chain compounds in both normal and infected groups was investigated by calculating the proportion of the peak area of each compound. The mean proportion is shown in Figure 7, and the detailed individual cellular toxin production (peak area) is listed in Table S2. The production of nine gambierones was significantly reduced by the infection (*p* < 0.05). Then, 44-methylgambierone was quantifiable in both control and infected algal extracts. The limits of detection (LOD) and quantification (LOQ) of analytical methods were determined by using signal-to-noise (S/N) ratios of 3:1 and 10:1. The LOD of 44-methylgambierone analysis was 0.066 ng/mL, and the LOQ was 0.22 ng/mL. The production levels of 44-methylgambierone in the control and infected groups were 3.2 ± 0.44 pg/cell and 2.1 ± 0.042 pg/cell, respectively. Unlike the gambierones, the alteration of cellular production of the detected polyol-polyene super-carbon chain compounds showed three patterns. For instance, six of these compounds (adducts: *m/z* 1001.76, 1019.77, 1036.8, 983.72, 939.721, and 1072.8) were significantly increased by the infection, while four of them (adducts: *m/z* 1415.06, 1063.55, 996.781, and 947.727) were decreased (*p* < 0.05). This phenomenon indicated that the influence of the infection on the polyol-polyene super-carbon chain compounds was complicated, which may involve biotransformation processes.

## 3. Discussion

It is quite certain that the pathogen in this study was *Pseudobodo* sp. based on the morphological characteristics and phylogenetic analysis. The SSU rRNA gene sequencing revealed that the pathogen *Pseudobodo* sp. was most closely related to *P. tremulans* (95.55%), and the ML tree showed the pathogen was placed in *Pseudobodo* clade with strong statistical supports for ML value (100) and Bayesian inference (1). In addition, the general morphological features of *Pseudobodo* sp. were consistent with reported *Pseudobodo* sp. strains [24,26,33], including similar egg- or pear-shaped bodies, two unequal flagella, the smooth posterior, and moving behavior. No mastigoneme was observed on the anterior flagellum, which is the same as *Pseudobodo* sp. KD51 but different from *P. tremulans* [22,26]. The *Pseudobodo* sp. in the present study possesses a cytostome, whereas Fenchel described *P. tremulans* as possessing a cytostome at the base of the anterior flagellum [33]. However, the location differs from that of the organism in the present study. Therefore, among all reported strains, *Pseudobodo* sp. in this study was the closest match to the *Pseudobodo* species reported by J. S. Parslow in terms of three morphological traits [25]. Namely, the body length of both strains was 2–4 μm, lacking mastigonemes on their anterior flagella, and they had a prominent lip on a cytostome. However, no infection abilities and molecular information of J. S. Parslow’s *Pseudobodo* sp. were found for further comparison.

In nature, bacteria not only play an important role in the dynamics of algal bloom stages, including initiation, growth, maintenance, and termination, but are also involved in the regulation of algae growth and metabolisms [34,35,36]. Bacteria are considered an inherent part of the physical environment of dinoflagellates both in vitro and in vivo, which is similar to the functional importance of gut microbiota in animals and humans, and changes in the bacterial community structure typically coincide with dysbiosis and alterations in host performance [37,38]. Bacteroidetes and Proteobacteria are dominant phyla in the culture of dinoflagellates [19,37,39]. In the present study, although Bacteroidetes and Proteobacteria remained the main phyla in both control and infected groups, the proportions of five genera belonging to these two phyla were significantly altered by the infection. *Labrenzia* sp. was reported to have the ability to produce dimethylsulfoniopropionate, which was likely involved in the stress tolerance of microalgae [40,41,42]. The increment of *Labrenzia* sp. in infected groups may support the phenomenon of infection tolerance of *G. balechii* 1123M1M10. The *Flammeovirgaceae* sp. are known to degrade complex polysaccharides, which is a main feature of invertebrate mucus [43]. The *Pseudobodo* sp. likely secretes mucus to attach the substratum or water film for feeding and reproduction (Video S1). High proportions of *Flammeovirgaceae* sp. in infected groups may indicate their mucus-degrading capabilities and their resistance potential for *Pseudobodo* sp. in algal culture. On the other hand, *Croceibacter* sp. and *Marinoscillum* sp. were significantly decreased by the infection. It is reported that *Marinoscillum* sp. has a positive correlation with toxin content in *G. balechii* cultures [19], suggesting the potential influence on algal toxin production by the infection.

No pathogenetic heterokont infecting any species of *Gambierdiscus* has been reported yet. Since the theca of *Gambierdiscus* is tough, the infective approach of *Pseudobodo* sp. to *G. balechii* remains to be further explored. During the process of infection, dinocyst-like cells were observed, which might be a natural response for *Gambierdiscus* to combat the stress caused by the infection [44]. The infection reduced the cardiotoxicity of zebrafish in the algal extract exposure experiment, which corresponded with the alteration of gambierones and certain super-carbon chain compounds. Gambierone and 44-methylgambierone have shown the ability to create a disequilibrium in voltage-gated sodium channels, which possess similar bioactivity to CTXs and may impact the heart and nervous systems of animals [14,31]. The systematic investigation of the variation of toxins produced by control and infected *G. balechii* shed light on the stressful effect of heterotrophic heterokonts on dinoflagellates, and by extension, their ecological relationship. In the previous study, gene content and transcriptomic studies of different *Gambierdiscus* species provided evidence to support the polyketide origin of these polyether ladder compounds [45,46]. Isotope labeling studies on okadaic acid proved that the cyclization of a polyepoxide precursor may be a general biosynthetic strategy for the construction of polycyclic ethers [47]. Our data suggested that the pathogen was likely to interfere with the biosynthetic route of polyketide, especially the cyclization steps, which was inferred from the observation that gambierones with multiple cyclizations decreased drastically.

## 4. Conclusions

In conclusion, we investigated the influence of a heterotrophic flagellate, *Pseudobodo* sp., on the highly toxic algal host *G. balechii* 1123M1M10. *Pseudobodo* sp. can influence the toxicity and toxin production of *G. balechii* 1123M1M10 and induce the alteration of the microbiome abundance in the algal culture. The detailed relationship between *Pseudobodo* sp., *G. balechii* 1123M1M10, and the bacterial community needs further research. Taken together, our findings shed light on the complex interactions surrounding dinoflagellates in nature.

## 5. Materials and Methods

### 5.1. Gambierdiscus Balechii 1123M1M10 and Pseudobodo sp. Cultivation

The *G. balechii* strain 1123M1M10 was collected from the Marakei Island, Republic of Kiribati (2°01′ N, 173°15′ E) reported in our previous study [48]. The strain was cultured in f/2-Si medium prepared with artificial seawater with a salinity of 30 at 22 ± 1 °C under a 12 h:12 h (light/dark) cycle with a light intensity of 70–90 mol photon m^−2^s^−1^. *Pseudobodo* sp. was found in the contaminated culture of *G. balechii* 1123M1M10 by observing a low algal growth rate and algal lytic phenomenon. The *Pseudobodo* sp. was propagated by co-culture with *G. balechii* 1123M1M10 under the same growth conditions described above.

### 5.2. Co-Culture Experiments

Free-living *Pseudobodo* sp. was harvested using Isopore membrane filters (10 μm pore size; Millipore, Dublin, Ireland) by gravity filtration to remove the host cells. For the infected groups, 10 mL of free-living *Pseudobodo* sp. was added to the new uninfected algal culture (90 mL). For the control groups, free-living *Pseudobodo* sp. was filtered by a 0.8 μm pore size membrane to remove *Pseudobodo* sp., and the filtrate (10 mL) was added to the new uninfected algal culture (90 mL). The experiments were conducted in three replicates. Cells were counted at the beginning and after 28 days of infection under culturing conditions.

### 5.3. Light Microscopy and Electron Microscopy

The light microscopic images and videos were captured using an inverted microscope (OLYMPUS CKX53, Tokyo, Japan) equipped with a TrueChrome Metrics HDMI Microscope Measuring digital camera. For electron microscopic observation, the infected *G. balechii* 1123M1M10 and pathogens were fixed in a 15 mL conical tube (Corning, Gilbert, AZ, USA) with 2% glutaraldehyde for 24 h at 4 °C. Then, the fixed samples were collected using polycarbonate membrane filters (3 μm pore size for the infected *G. balechii* 1123M1M10, and 0.22 μm for *Pseudobodo* sp.; Millipore, Ireland). Desalting and dehydration in a sterilized artificial seawater and ethanol series were conducted, respectively. Samples were critical point dried in liquid CO_2_ using a CPD 030 (BAL-TEC, Tokyo, Japan). Then, they were subsequently glued to SEM stubs with carbon tape, sputter-coated with platinum, and examined with an ESEM (model Quattro S, Thermo Fisher, Waltham, MA, USA) scanning electron microscope operating at 5 kV or 10 kV.

### 5.4. DNA Extraction, PCR Amplification, and Sequencing for Pseudobodo sp.

Free-living *Pseudobodo* sp. was harvested using the above-mentioned method and then concentrated by filters (0.22 μm pore size; Millipore, Ireland). DNA extraction was conducted following the reported protocol [49]. Polymerase chain reaction (PCR) amplifications were performed with primers EuKB and 342-F for the small subunit (SSU) rRNA gene [26,50]. PCRs were conducted in 20 μL of reaction solution containing 80 ng of DNA extracts as a template, 10 μL SapphireAmp Fast PCR Master Mix (TAKARA, Kusatsu, Japan), and 0.4 μL of each primer (10 pmol/mL). PCRs were run in an automated thermocycler (Bio-Rad C1000 Touch Cycler W/48W, Bio-RAD, Hercules, CA, USA) under the following conditions: the initial denaturing step was 94 °C for 1 min, then 30 cycles of 5 s at 98 °C, 5 s at 60 °C, and 15 s at 72 °C, followed by a final extension step of 10 min at 72 °C. Amplified PCR products were loaded to 1.5% agarose gel to verify the success of PCR and purified with a PCR purification kit (TAKARA, Kusatsu, Japan). The PCR primers and internal primers (F1: 5′-TGC CTT GAA TAC ATT AGC ATG GA-3′) were used to sequence the complete SSU rRNA gene. Sequences were aligned and assembled using SnapGene 4.2.4 software (Insightful Science, San Diego, CA, USA).

### 5.5. Pseudobodo sp. SSU rRNA Gene Alignment and Phylogenetic Analyses

The obtained sequence was primarily aligned with related sequences from the NCBI Genbank database using the ClustalW [51] portion of BioEdit 7.2.5 (North Carolina State University, Raleigh, NC, USA). All aligned nuclear rRNA gene sequences were trimmed to the same length, and the gaps were deleted. For maximum likelihood (ML) phylogenetic tree analysis, the best-fit model (GRT+I+G) for the MEGA X [52] settings was selected from 24 tested models using MrModelTest2.3. Bootstrap values (branch support) were obtained from 1000 replicates, and values >50 were indicated at each branch node. For Bayesian inference (BI) analysis, the best-fit model (GRT+I+G) was selected for the settings of MrBayes 3.2.1 [53]. The Markov Chain Monte Carlo (MCMC) process was set at four chains and 5,000,000 generations were performed. The sampling frequency was 100 generations. Following analysis, the standard deviation of frequencies was confirmed to be <0.01. Bayesian posterior probabilities (BI) > 0.50 were indicated at each branch node. The SSU rRNA gene sequence of *Pseudobodo* sp. in this study was deposited in Gen-Bank (accession number: ON076887).

### 5.6. High-Throughput Sequencing of Microbiome 16S rRNA Gene Sequencing

Five-milliliter samples were collected in triplicate from both infected and control groups of *G. balechii* 1123M1M10 cultures. Then, these samples were filtered onto 0.22 µm polycarbonate membranes (Millipore, Ireland), and then transferred into a 2 mL microcentrifuge tube and immersed in 800 μL of DNA extraction buffer (100 mM of Tris-HCl with pH 8, 100 mM of Na2-EDTA, 100 mM of sodium phosphate with pH 8, 1.5 M NaCl, and 1% CTAB) and stored at −80 °C until further processing. DNA extraction followed the reported protocol [49]. The V3-V4 region of the 16S rRNA gene was amplified from each culture using primers of 341F (forward primer, 5′-CCTACGGGNGGCWGCAG-3′) and 805R (reverse primer 5′-GACTACHVGGGTATCTAATCC-3′). PCR was carried out as follows: 95 °C for 3 min, followed by 5 cycles of denaturing at 95 °C (30 s), annealing at 45 °C (30 s), and elongation at 72 °C (30 s), then 20 cycles of denaturing at 95 °C (30 s), annealing at 55 °C (30 s), elongation at 72 °C (30 s), and a final extension at 72 °C for 5 min. The PCR reaction used 2×Hieff^®^ Robust PCR Master Mix (Yeasen, 10105ES03, Shangai, China). Before sequencing, the DNA concentration of each PCR product was determined using a Qubit^®^ 4.0 Green double-stranded DNA assay, and quality control was performed using a bioanalyzer (Agilent 2100, Santa Clara, CA, USA). The retrieved DNA was purified, uniquely barcoded for each sample, and pooled for sequencing on the Illumina MiSeq system (Illumina MiSeq, San Diego, CA, USA).

### 5.7. Assessment of Cardiotoxicity of Algal Extracts in Zebrafish

All animal experiments were approved by the Institutional Animal Care and Use Committee (IACUC) of Zhejiang University (Hangzhou, Zhejiang, China). The wild-type (WT) AB zebrafish (*Danio rerio*) strain was used in this study. Zebrafish maintenance and the preparation of zebrafish embryos followed the standard procedure [54]. Exposure to algal extracts was performed on fresh zebrafish eggs within 0.5 h post-fertilization (hpf). In a well of a 24-well plate, three fertilized eggs were exposed to 25, 250, 1667, and 8333 cells eq·mL^−1^ of algal extracts used with a 1.2 mL volume. After 72 h exposure, the heart rates (beats per minute, bpm, *n* = 12) and pericardial area (μm^2^, *n* = 6) were counted and measured, respectively.

### 5.8. Sample Collection and Treatment for HPLC-MS/MS Analysis

The sample collection and treatment procedures for HPLC-MS/MS analysis were performed following the protocols described in our previous work [11]. After extraction, the lysate was evaporated under a gentle stream of high-purity nitrogen and then underwent solvent partition using dichloromethane (DCM) and 60% aqueous methanol, repeated three times, resulting in two fractions (DCM layer and 60% MeOH layer). The final extract of each fraction was concentrated and redissolved in 4 mL of methanol. The samples were diluted two times with methanol and filtered through a 0.2 µm polypropylene filter (GL Sciences, Tokyo, Japan) for instrumental analysis.

### 5.9. Toxin Standards

Gambierone and 44-methylgambierone were purchased from Laboratorio CIFGAS.A. (Lugo, Spain). P-CTX-4A and 2,3-dihydroxy CTX-3C were provided by Dr. Mireille Chinain from the Institut Louis Malardé’s bank of standards (ILM, Papeete, Tahiti, French Polynesia). Maitotoxin1 (MTX1) was purchased from Wako Chemicals GmbH (Neuss, Germany).

### 5.10. Instrumental Analysis

The instrumental methods for analyzing algal toxins followed our previous study [11]. The non-target analysis was performed using high-performance liquid chromatography-tandem mass spectrometry (HPLC-MS/MS) consisting of an Agilent 1290 UPLC system (Agilent, Palo Alto, CA, USA) and a Sciex X500R QTOF system (AB Sciex, Foster City, CA, USA) operating in the positive electrospray ionization information-dependent acquisition (IDA) mode. The determination of 44-methylgambierone was performed using an Agilent 1290 Infinity ultra-performance liquid chromatograph (Palo Alto, CA, USA) interfaced with a Sciex 5500 QTRAP mass spectrometer (Foster City, CA, USA) operating in the negative electrospray ionization multiple reaction monitoring (MRM) mode.

### 5.11. Untargeted Toxin Profiling and Relative Quantification

Raw data of samples and standards were converted into the mzXML format using ProteoWizard MSConvert freeware. During the conversion process, a 64-bit encoding precision and Zlib compression were selected. Then, the mzXML files were uploaded to the UCSD GNPS WinSCP server (http://gnps.ucsd.edu, 12 July 2023) and investigated via the METABOLOMICS-SNETS-V2 workflow [39] with the following parameters: The data were filtered by removing all MS/MS fragment ions within ±17 Da of the precursor *m*/*z*. Parent mass tolerance was set to 2 Da and fragment ion mass tolerance to 0.1 Da. The edges were filtered to have a cosine score above 0.5 and more than 4 matched peaks. Further, edges between two nodes were kept in the network if and only if each of the nodes appeared in each other’s respective top 10 most similar nodes. Finally, the maximum size of a molecular family was set to 100. Data were visualized via Cytoscape (ver. 3.8.0) and the networks were determined using the online tool (https://gnps.ucsd.edu/ProteoSAFe/status.jsp?task=75ee1cc333154e74a31597e7aaa014cd, 28 July 2023).

According to the results of molecular networking (MN), a list of MS1 was created. The peak area was calculated to compare the target components in the infected and control groups. Peak areas were normalized to the cell density (Table S1). The peak list was extracted from the raw QTOF-MS data in the SCIEX OS. The following parameters were set for initial filtering: (1) S/N > 3 and (2) intensity > 100.

### 5.12. Data Analysis

A Student’s *t*-test was used to determine differences between two groups with normally distributed data, while the one-way ANOVA with Duncan’s tests was applied for the comparison among multiple groups at a significance level of *p* < 0.05. Graphs were plotted by R 4.2, Origin 2021, and Cytoscape 3.8. Images were processed by Illustrator 2022.

## Data Availability

The data presented in this study are available on request from the corresponding authors.

## Supplementary Materials

The following supporting information can be downloaded at: https://www.mdpi.com/article/10.3390/toxins15110657/s1, Figure S1: MS/MS spectra of gambierone analogs. The specific neutral losses and fragments of gambirones are shown in blue, and mass errors of specific fragments are shown in red; Figure S2: MS/MS spectra of polyol-polyene super-carbon-chain compounds; Table S1: Total algal cell number in each group; Table S2: Cellular production of individual toxins in the control and infected group; Video S1: The morphology, moving behavior, and life cycle of the heterotrophic bicosoecid flagellate *Pseudobodo* sp. (400 times magnification and 4 times speed).
