# Supplementary material for: Characterizing the Influence of a Heterotrophic Bicosoecid Flagellate Pseudobodo sp. on the Dinoflagellate Gambierdiscus balechii
Source: Toxins (Basel). 2023 Nov 14;15(11):657. doi: 10.3390/toxins15110657 (PMC10674679; doi:10.3390/toxins15110657)
Supplement: Supplementary file 1 [file toxins-15-00657-s001.zip › toxins-2680644-supplementary.pdf]

toxins

MDPI

Article

Characterizing the Influence of a Heterotrophic Bicosoecid Flagellate *Pseudobodo* sp. on the Dinoflagellate *Gambierdiscus balechii*

Table S1. Total algal cell number in each group.

Groups	Control groups			Infected groups		
	A1	A2	A3	B1	B2	B3
Cell Number	$6.52×10^5$	$6.00×10^5$	$5.93×10^5$	$3.08×10^5$	$2.38×10^5$	$2.42×10^5$

Table S2. Cellular production of individual toxins in the control and infected group.

Observed *m*/*z*	Retention time (min)	Peak area (mean ± SD)		Significant difference
		Control group	Infected group	
**1039.5041**	**6.55**	278.19 ± 33.50	172.54 ± 10.00	$p < 0.05$
1041.5166	6.28	214.02 ± 30.77	145.17 ± 8.89	$p < 0.05$
1021.4921	6.57	47.52 ± 3.71	24.76 ± 2.38	$p < 0.05$
1023.5066	6.28	48.74 ± 5.29	29.57 ± 3.99	$p < 0.05$
926.4497	3.65	57.37 ± 3.94	35.37 ± 2.03	$p < 0.05$
980.5669	5.61	61.09 ± 9.15	38.71 ± 0.80	$p < 0.05$
990.5030	3.96	30.09 ± 4.06	19.10 ± 1.89	$p < 0.05$
943.5472	6.28	34.40 ± 2.78	20.79 ± 1.39	$p < 0.05$
944.4608	3.67	23.97 ± 2.57	15.07 ± 1.06	$p < 0.05$
958.4905	5.20	96.47 ± 27.09	52.00 ± 0.26	$p > 0.05$
1154.5658	6.21	65.80 ± 23.04	26.30 ± 0.35	$p > 0.05$
1060.5256	4.86	19.52 ± 3.40	13.99 ± 2.48	$p > 0.05$
1042.5164	5.47	14.99 ± 2.43	11.20 ± 0.03	$p > 0.05$
1171.5919	6.21	18.51 ± 7.09	8.23 ± 0.64	$p > 0.05$
1001.7577	6.42	50.74 ± 13.13	76.03 ± 3.87	$p < 0.05$
1019.7697	6.40	86.27 ± 22.50	135.74 ± 5.80	$p < 0.05$
1036.7966	6.42	12.48 ± 3.17	20.94 ± 0.79	$p < 0.05$
1063.5001	6.25	15.78 ± 1.96	11.60 ± 1.16	$p < 0.05$
983.7480	6.41	40.41 ± 10.42	61.28 ± 3.58	$p < 0.05$

947.7255	21.67	13.80 ± 3.52	0.22 ± 0.38	$p < 0.05$
996.7793	21.70	9.48 ± 2.21	ND	$p < 0.05$
1415.0502	28.11	59.21 ± 3.64	37.78 ± 10.55	$p < 0.05$
939.7218	30.41	81.46 ± 8.07	117.89 ± 12.10	$p < 0.05$
1072.7972	31.76	602.14 ± 126.88	834.62 ± 69.50	$p < 0.05$
1092.8602	7.77	11.59 ± 2.57	9.26 ± 0.75	$p > 0.05$
1076.8276	6.88	67.25 ± 17.12	51.62 ± 12.01	$p > 0.05$
1075.8319	7.81	49.51 ± 11.33	40.81 ± 2.43	$p > 0.05$
1080.8220	6.15	26.47 ± 3.99	32.70 ± 3.28	$p > 0.05$
1057.8229	7.72	51.63 ± 12.74	39.56 ± 2.35	$p > 0.05$
1027.7753	6.72	322.48 ± 82.79	311.38 ± 29.06	$p > 0.05$
1041.7906	6.95	173.89 ± 42.88	128.86 ± 15.02	$p > 0.05$
1059.8024	6.90	54.38 ± 14.35	38.77 ± 5.10	$p > 0.05$
1045.7870	6.07	181.25 ± 41.68	187.34 ± 13.76	$p > 0.05$
1029.7904	7.20	278.78 ± 78.96	316.37 ± 20.35	$p > 0.05$
1047.8019	7.22	163.27 ± 49.55	198.66 ± 17.04	$p > 0.05$
1064.8277	7.29	143.25 ± 44.94	177.59 ± 15.86	$p > 0.05$
895.6585	26.38	63.86 ± 5.34	58.64 ± 10.08	$p > 0.05$
953.6993	25.09	13.26 ± 2.55	17.43 ± 2.37	$p > 0.05$
1055.7699	31.75	585.22 ± 95.01	703.84 ± 80.58	$p > 0.05$
1069.7499	26.39	91.60 ± 4.04	86.80 ± 20.55	$p > 0.05$
1086.7762	26.30	94.97 ± 6.53	98.63 ± 24.83	$p > 0.05$
543.8892	26.36	308.10 ± 20.12	287.73 ± 48.96	$p > 0.05$

Note: ND = not detected.

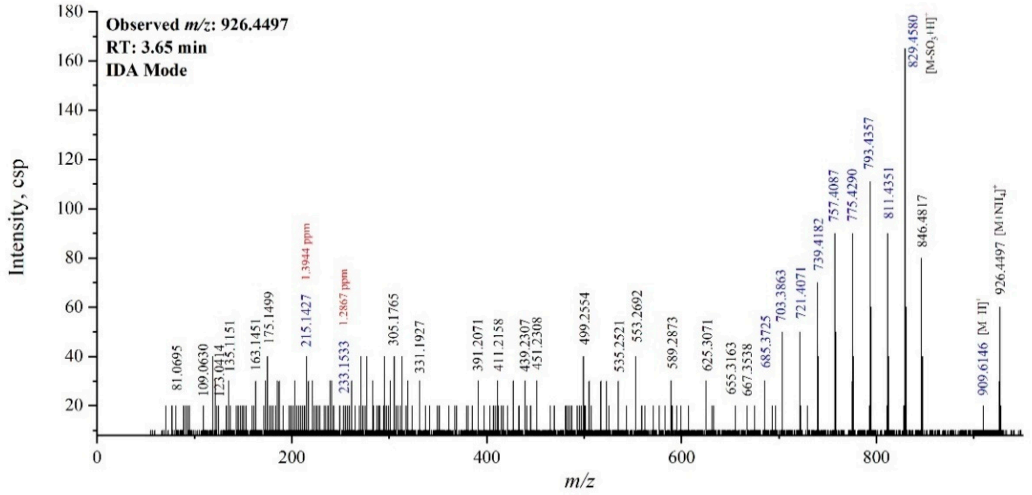
Observed m/z: 926.4497
RT: 3.65 min
IDA Mode
Intensity, csp
m/z
81.0695
109.0630
123.0414
135.1151
163.1451
175.1499
215.1427
1.3944 ppm
233.1533
1.2867 ppm
305.1765
331.1927
391.2071
411.2158
439.2307
451.2308
499.2554
535.2521
553.2692
589.2873
625.3071
655.3163
667.3538
685.3725
703.3863
721.4071
739.4182
757.4087
775.4290
793.4357
811.4351
829.4580 [M-SO3+H]+
846.4817
909.6146 [M+H]+
926.4497 [M+NH4]+

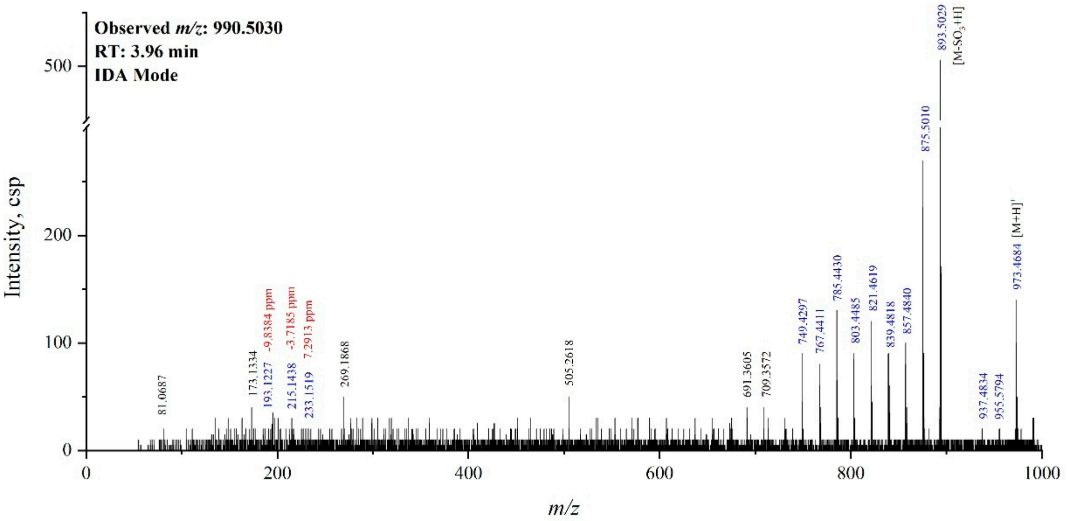
Observed m/z: 990.5030
RT: 3.96 min
IDA Mode
Intensity, csp
m/z
81.0687
173.1334
193.1227
-9.8384 ppm
215.1438
-3.7185 ppm
233.1519
7.2913 ppm
269.1868
505.2618
691.3605
709.3572
749.4297
767.4411
785.4430
803.4485
821.4619
839.4818
857.4840
875.5010
893.5029 [M-SO3+H]+
937.4834
955.5794
973.4684 [M+H]+

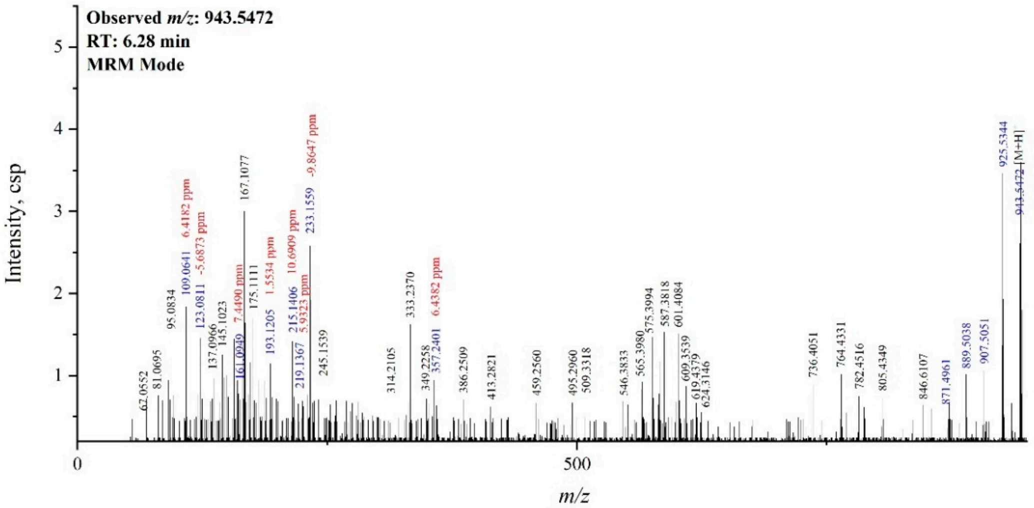
Observed m/z: 943.5472
RT: 6.28 min
MRM Mode
Intensity, csp
m/z
67.0552
81.0695
95.0834
109.0641
6.4182 ppm
123.0811
-5.6873 ppm
137.0966
145.1023
161.0949
7.4490 ppm
167.1077
175.1111
193.1205
1.5534 ppm
215.1406
10.6909 ppm
219.1367
5.9323 ppm
233.1559
-9.8647 ppm
245.1539
314.2105
333.2370
349.2258
357.2401
6.4382 ppm
386.2509
413.2821
459.2560
495.2960
509.3318
546.3833
565.3980
575.3994
587.3818
601.4084
609.3539
619.4379
624.3146
736.4051
764.4331
782.4516
805.4349
846.6107
871.4961
889.5038
907.5051
925.5344
943.5472 [M+H]+

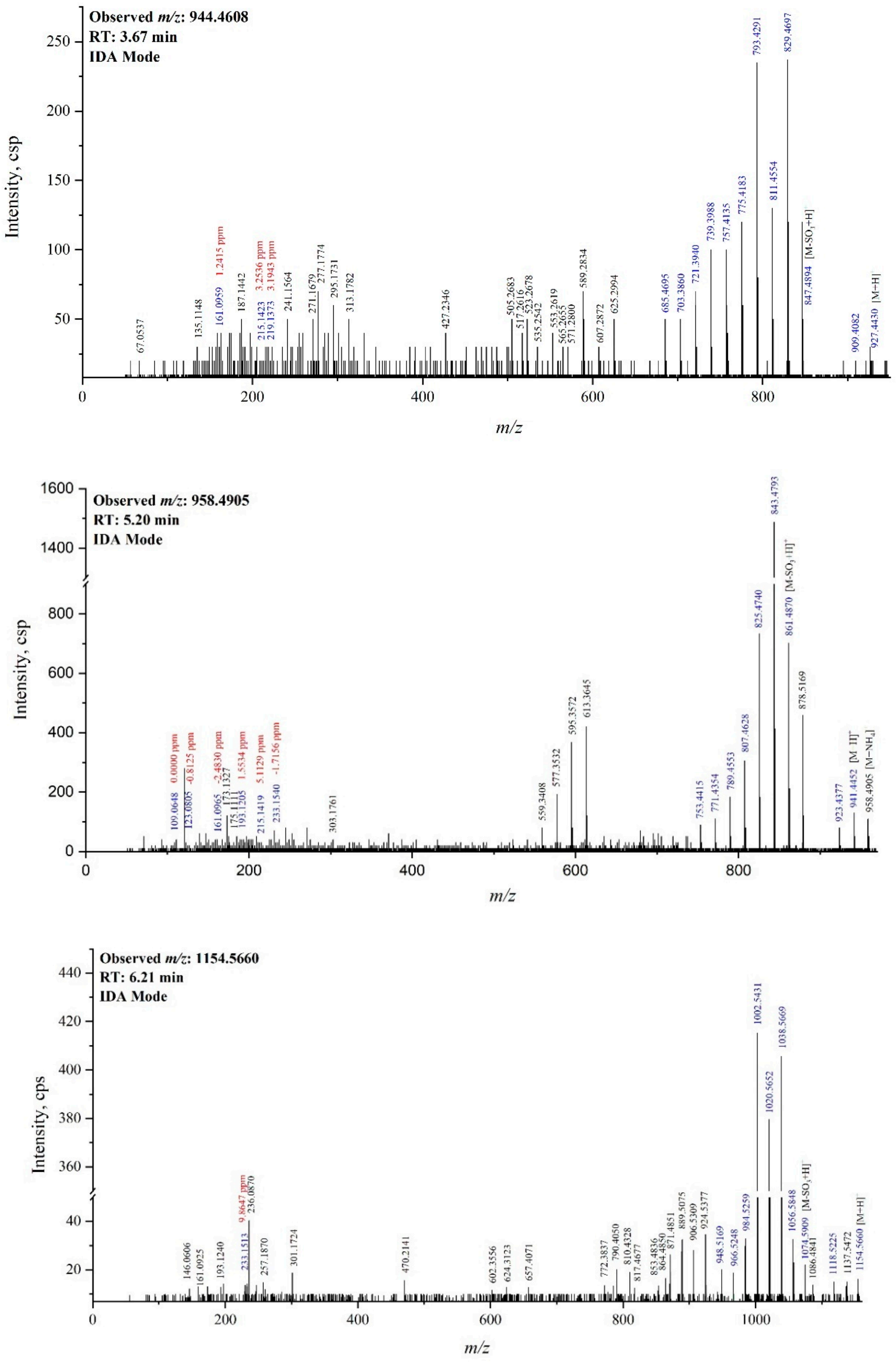
Observed m/z: 944.4608
RT: 3.67 min
IDA Mode
Intensity, csp
m/z
793.4291
829.4697
811.4554
775.4183
757.4135
739.3988
721.3940
703.3860
685.4695
847.4894 [M-SO3+H]
909.4082
927.4430 [M−H]
Observed m/z: 958.4905
RT: 5.20 min
IDA Mode
Intensity, csp
m/z
843.4793
861.4870 [M-SO3+H]+
825.4740
878.5169
807.4628
613.3645
595.3572
577.3532
559.3408
Observed m/z: 1154.5660
RT: 6.21 min
IDA Mode
Intensity, cps
m/z
1002.5431
1038.5669
1020.5652
1056.5848 [M-SO3+H]+
1154.5660 [M−H]+

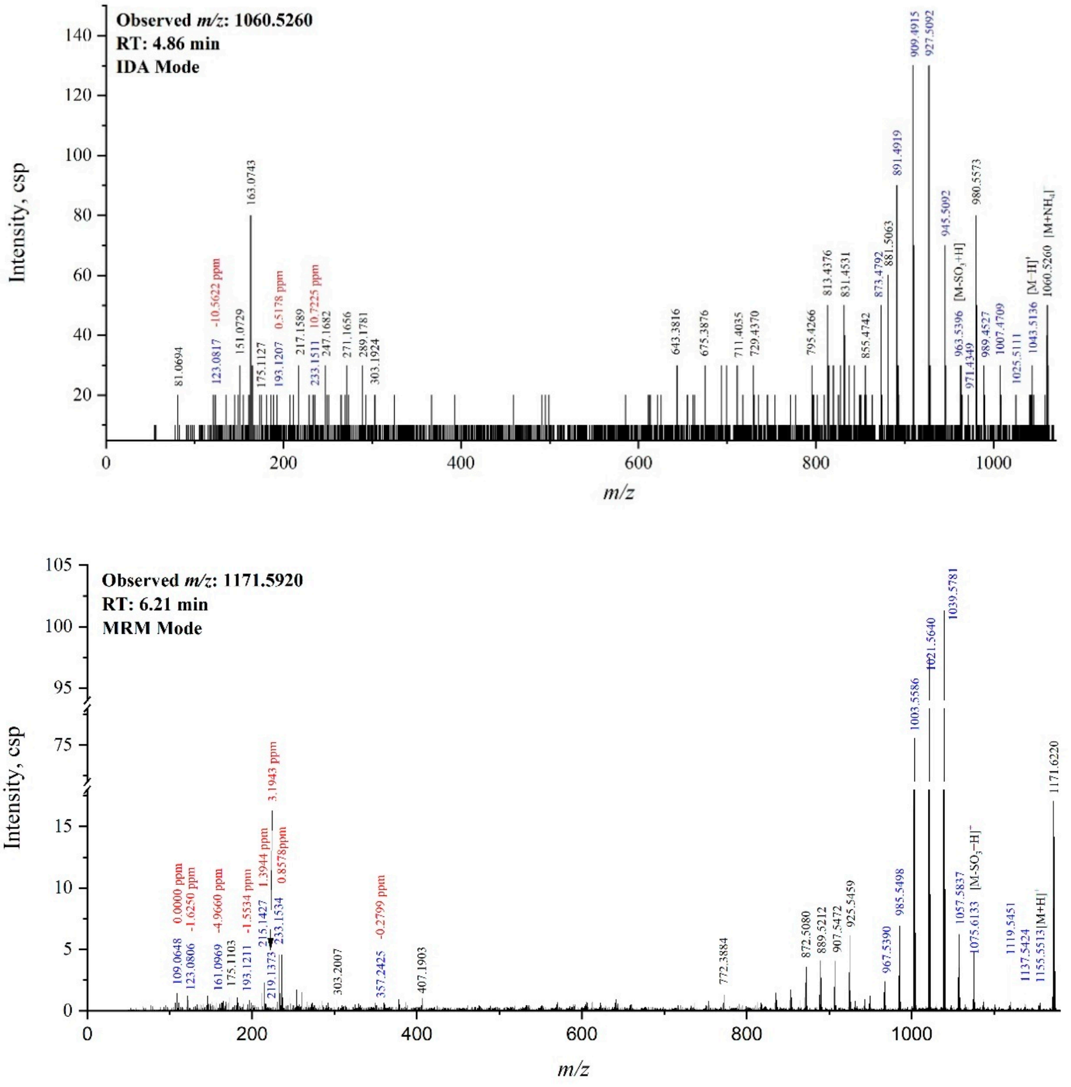

Figure S1. MS/MS spectra of gambierone analogues. The specific neutral losses and fragments of gambirones were shown in blue, and mass errors of specific fragments were shown in red.

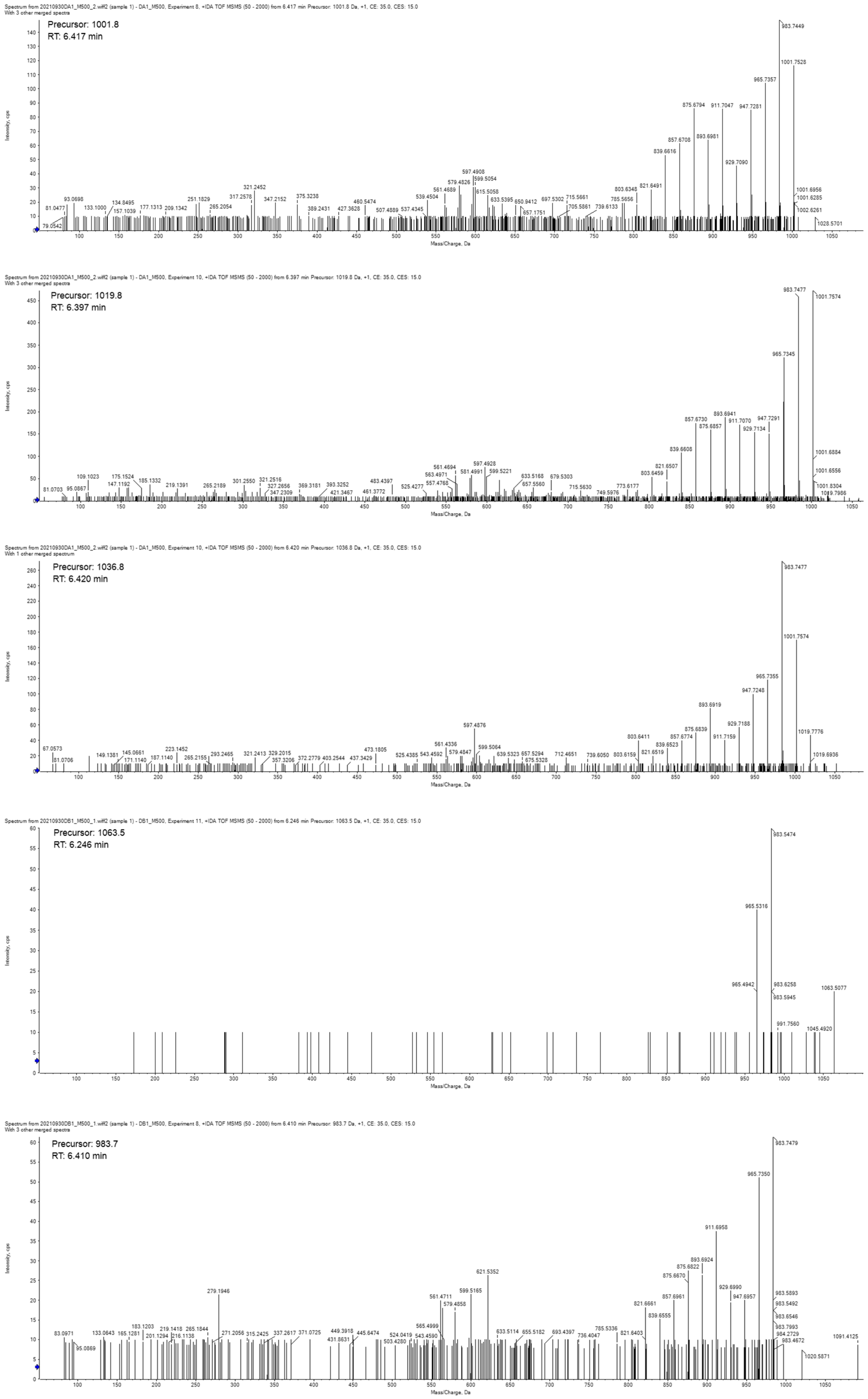
Precursor: 1001.8
RT: 6.417 min
Precursor: 1019.8
RT: 6.397 min
Precursor: 1036.8
RT: 6.420 min
Precursor: 1063.5
RT: 6.246 min
Precursor: 983.7
RT: 6.410 min
Mass/Charge, Da
Intensity, cps

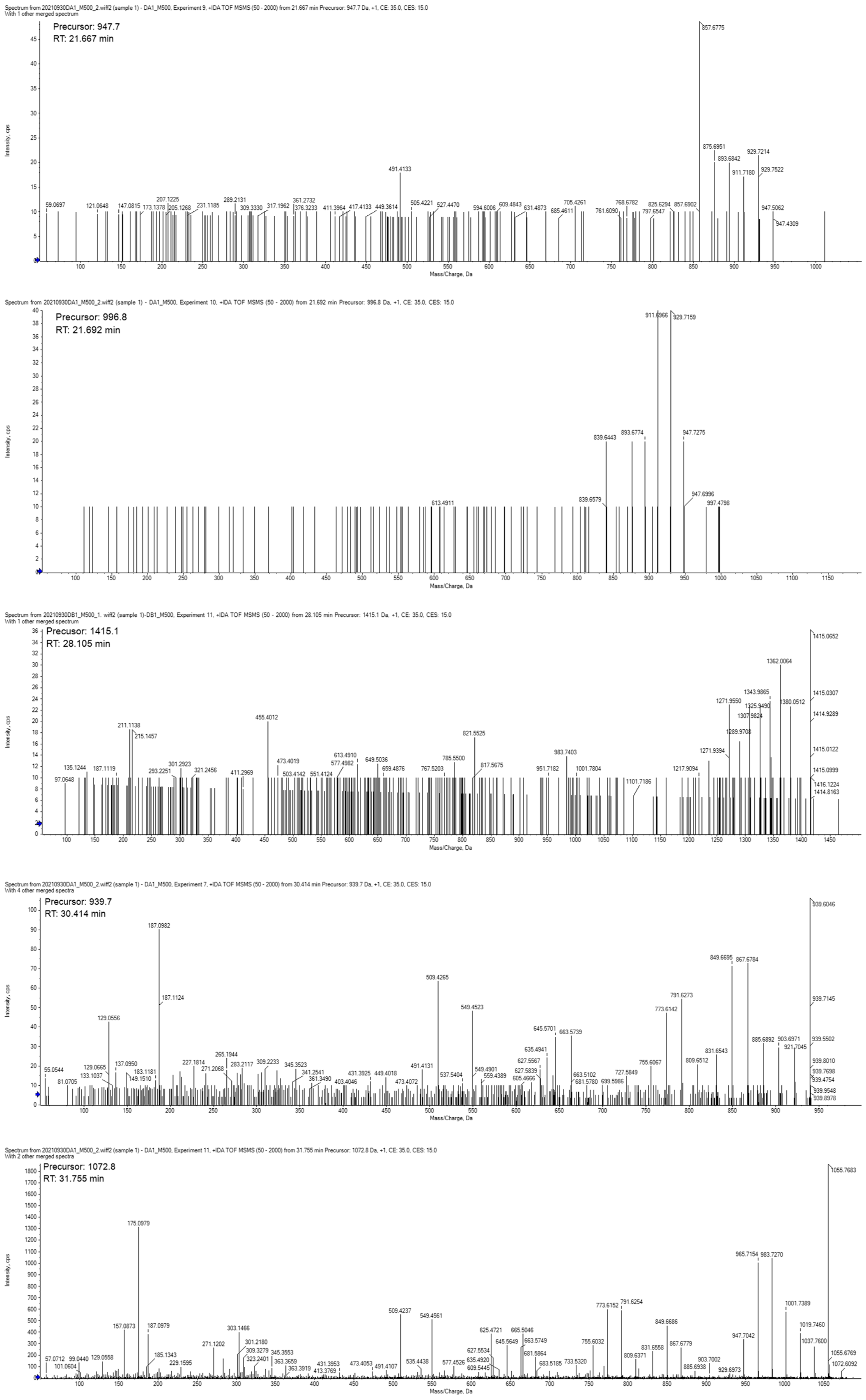

Spectrum from 20210930DA1_M500_2.wiff2 (sample 1) - DA1_M500, Experiment 9, +IDA TOF MSMS (50 - 2000) from 21.667 min Precursor: 947.7 Da, +1, CE: 35.0, CES: 15.0
With 1 other merged spectrum
Precursor: 947.7
RT: 21.667 min
Intensity, cps
Mass/Charge, Da
Spectrum from 20210930DA1_M500_2.wiff2 (sample 1) - DA1_M500, Experiment 10, +IDA TOF MSMS (50 - 2000) from 21.692 min Precursor: 996.8 Da, +1, CE: 35.0, CES: 15.0
Precursor: 996.8
RT: 21.692 min
Intensity, cps
Mass/Charge, Da
Spectrum from 20210930DB1_M500_1. wiff2 (sample 1)-DB1_M500, Experiment 11, +IDA TOF MSMS (50 - 2000) from 28.105 min Precursor: 1415.1 Da, +1, CE: 35.0, CES: 15.0
With 1 other merged spectrum
Precursor: 1415.1
RT: 28.105 min
Intensity, cps
Mass/Charge, Da
Spectrum from 20210930DA1_M500_2.wiff2 (sample 1) - DA1_M500, Experiment 7, +IDA TOF MSMS (50 - 2000) from 30.414 min Precursor: 939.7 Da, +1, CE: 35.0, CES: 15.0
With 4 other merged spectra
Precursor: 939.7
RT: 30.414 min
Intensity, cps
Mass/Charge, Da
Spectrum from 20210930DA1_M500_2.wiff2 (sample 1) - DA1_M500, Experiment 11, +IDA TOF MSMS (50 - 2000) from 31.755 min Precursor: 1072.8 Da, +1, CE: 35.0, CES: 15.0
With 2 other merged spectra
Precursor: 1072.8
RT: 31.755 min
Intensity, cps
Mass/Charge, Da

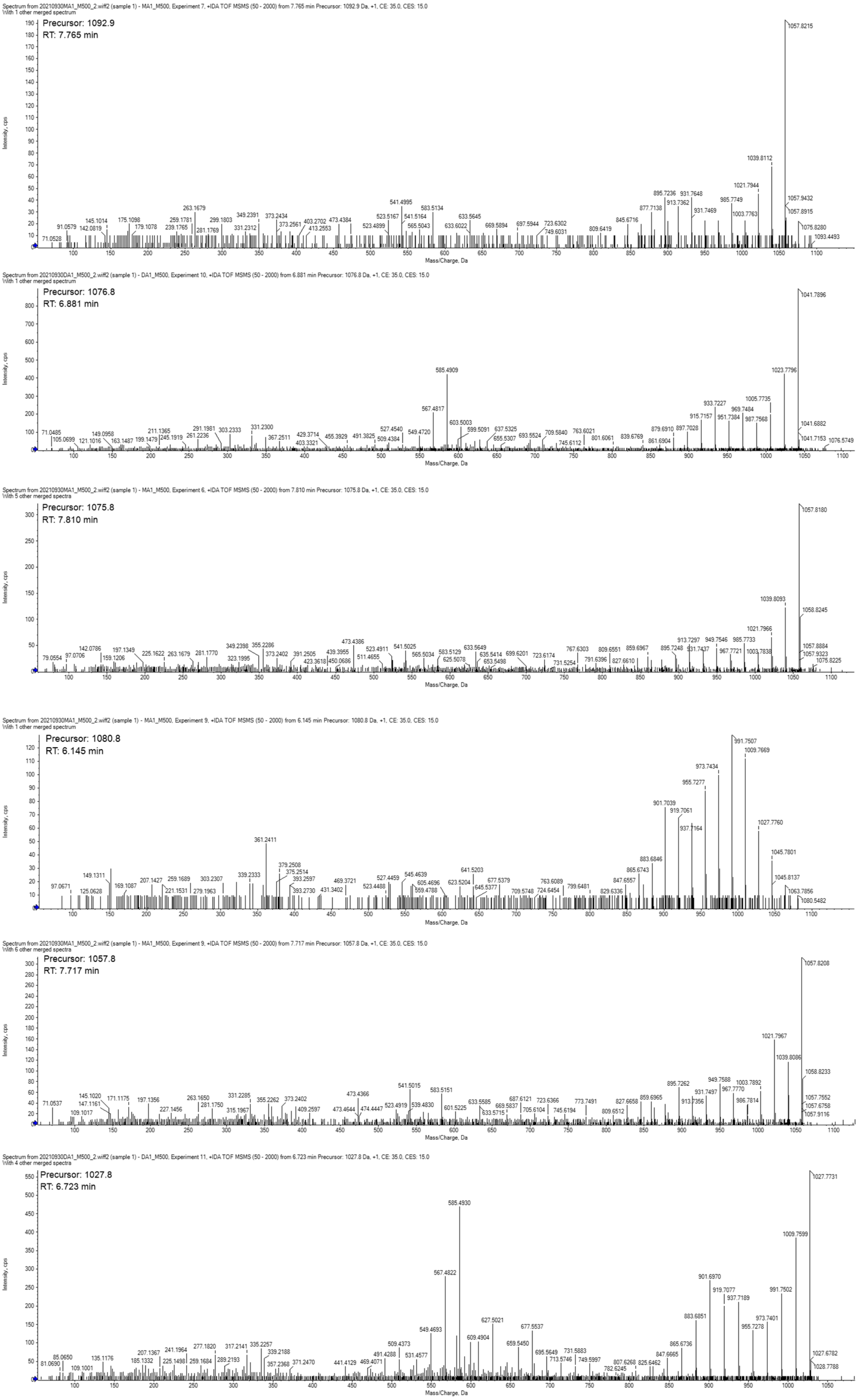
Precursor: 1092.9
RT: 7.765 min
Precursor: 1076.8
RT: 6.881 min
Precursor: 1075.8
RT: 7.810 min
Precursor: 1080.8
RT: 6.145 min
Precursor: 1057.8
RT: 7.717 min
Precursor: 1027.8
RT: 6.723 min
Intensity, cps
Mass/Charge, Da

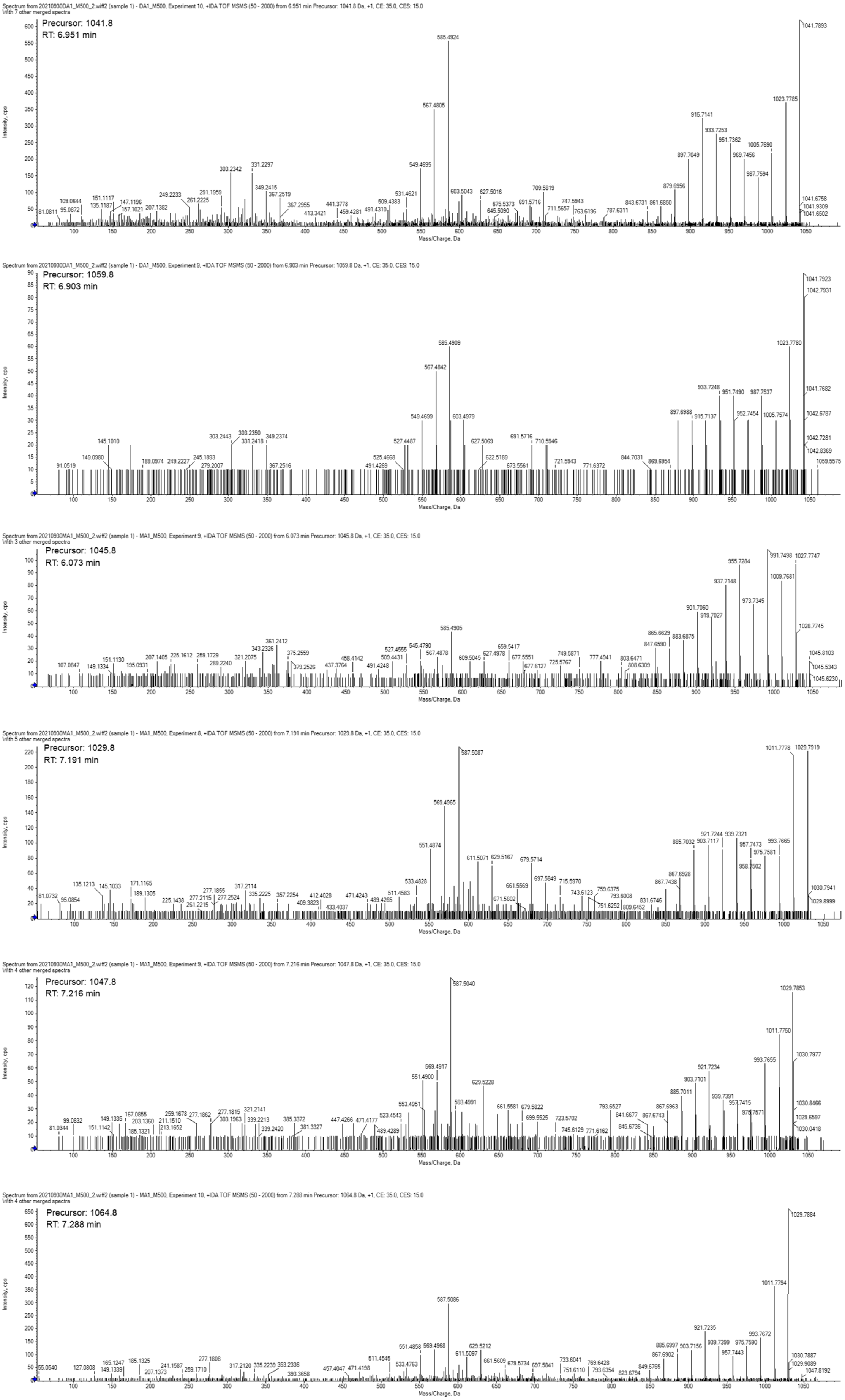
Precursor: 1041.8
RT: 6.951 min
Precursor: 1059.8
RT: 6.903 min
Precursor: 1045.8
RT: 6.073 min
Precursor: 1029.8
RT: 7.191 min
Precursor: 1047.8
RT: 7.216 min
Precursor: 1064.8
RT: 7.288 min
Intensity, cps
Mass/Charge, Da

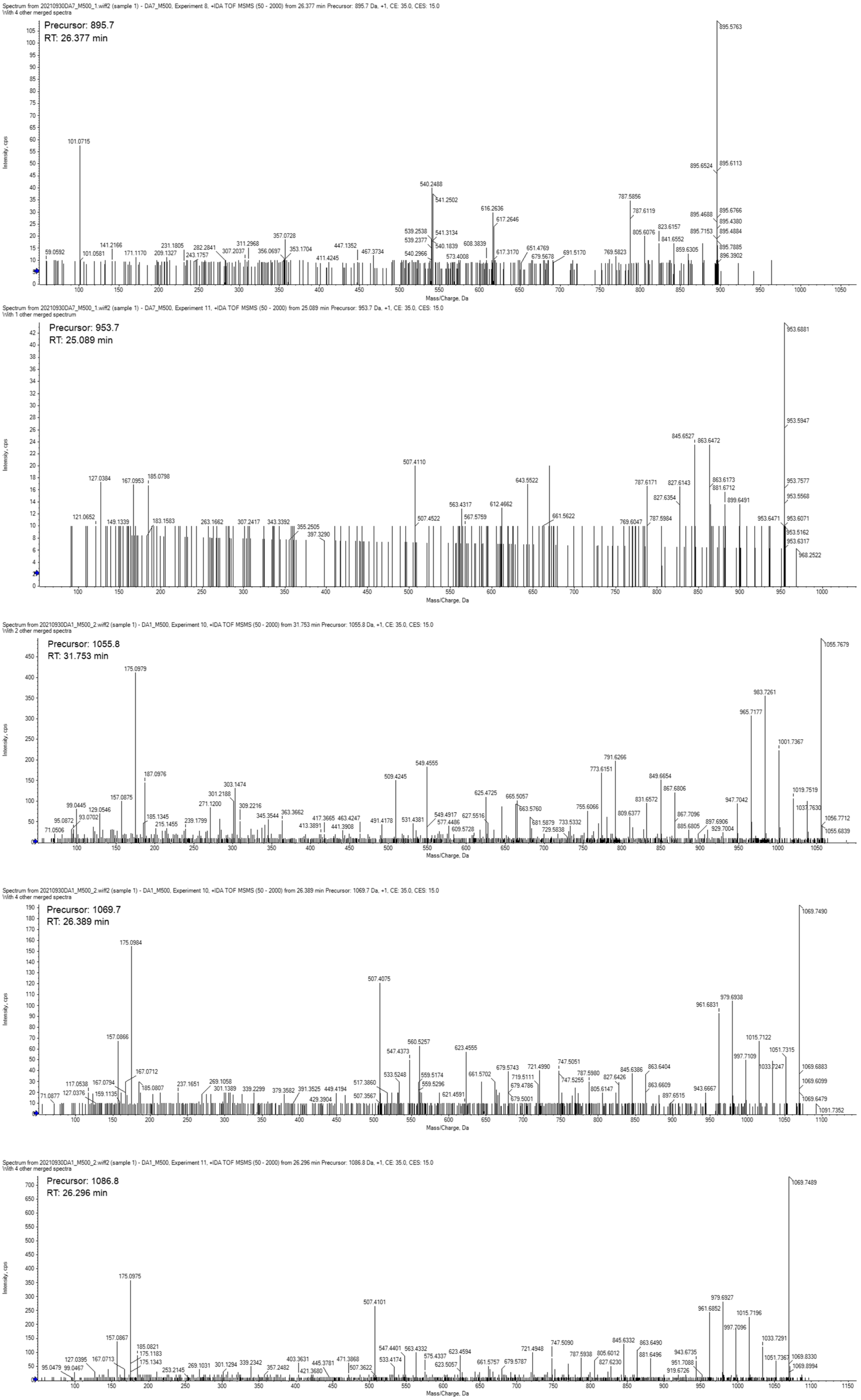
Precursor: 895.7
RT: 26.377 min
Intensity, cps
Mass/Charge, Da
Precursor: 953.7
RT: 25.089 min
Intensity, cps
Mass/Charge, Da
Precursor: 1055.8
RT: 31.753 min
Intensity, cps
Mass/Charge, Da
Precursor: 1069.7
RT: 26.389 min
Intensity, cps
Mass/Charge, Da
Precursor: 1086.8
RT: 26.296 min
Intensity, cps
Mass/Charge, Da

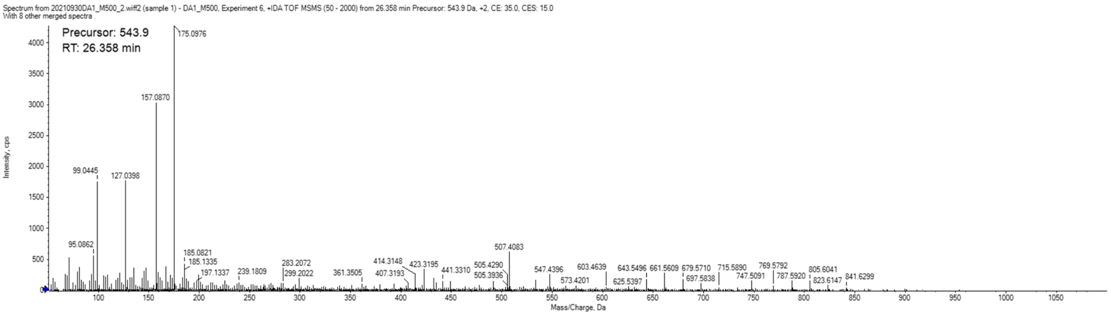

Figure S2. MS/MS spectra of polyol-polyene super-carbon-chain compounds.
